# Clinical Value of Paraspinal Muscle Quality Changes in Risk Assessment of Osteoporotic Vertebral Compression Fracture

**DOI:** 10.2174/0115734056469276260630201438

**Published:** 2026-07-09

**Authors:** Jiaqiu Jiang, Haolei Wang, Bo He, Jiaxin Chen, Zhengguang Zhang, Lei Yang, Yilong Huang

**Affiliations:** 1 Department of Medical Imaging, The First Affiliated Hospital of Kunming Medical University, 650032 Kunming, China; 2 Department of Medical Imaging, the Mental Hospital of Yunnan Province, 650000 Kunming, China

**Keywords:** Osteoporotic vertebral compression fracture, Paraspinal muscles, Fat infiltration, Fracture risk assessment, Poor prognosis, Elderly population

## Abstract

**Background:**

Osteoporotic vertebral compression fractures (OVCFs) are often underestimated by conventional tools. Paraspinal muscle quality changes, especially reduced cross-sectional area (CSA) and increased fatty infiltration (FI), may provide additional clinical value for OVCF risk.

**Materials and Methods:**

We retrospectively enrolled 258 osteoporosis patients aged ≥50 years. After propensity score matching, 129 patients with vertebral fractures and 129 without fractures were included. Demographics, BMD T-scores, and FRAX scores were collected. Paraspinal muscle CSA and FI at the L3 level were quantified on axial T2WI using ImageJ. Group comparisons and multivariable logistic regression were performed, and diagnostic performance was evaluated using ROC analysis.

**Results:**

Compared with the non-fracture group, fracture patients had significantly lower weight, BMI, and CSA but higher FRAX scores and FI. FI remained elevated in the fracture group across all BMI subgroups (*P*<0.05), whereas CSA reduction was significant only in the normal-weight subgroup (*P*=0.001). FI was an independent risk factor for OVCF (OR=1.091, 95% CI: 1.041–1.144; *P*<0.001). FI showed higher discrimination (AUC=0.702) than BMD T-score (AUC=0.517) and FRAX (AUC=0.592). The combined model achieved the best performance (AUC=0.776; sensitivity 54.4%, specificity 89.7%; all *P*<0.05).

**Discussion:**

Paraspinal FI, a marker of muscle quality deterioration, provides complementary risk information beyond BMD and FRAX and may help identify high-risk individuals who are underestimated by traditional assessments.

**Conclusion:**

Paraspinal muscle FI is an independent risk factor for OVCF and improves prediction when added to traditional tools.

## 1 INTRODUCTION

Osteoporotic Vertebral Compression Fractures (OVCFs) are common and serious complications of osteoporosis, with incidence steadily increasing due to global population aging [1]. With the global population aging, osteoporosis and related fragility fractures have become a major public health concern worldwide and a leading cause of disability and mortality in the elderly population [2]. OVCFs are particularly clinically challenging because of their insidious onset, frequent underdiagnosis, poor prognosis, and high risk of refracture [3]. Therefore, early identification of high-risk individuals is crucial for improving patient outcomes and reducing healthcare burdens.

The primary cause of OVCFs is the osteoporosis-induced deterioration of bone strength (including both bone mass and quality), and its pathological mechanisms are the decrease in bone mineral content and the destruction of bone microstructure [4]. Currently, the risk assessment of OVCF mainly relies on bone mineral density (BMD) measured by dual-energy X-ray absorptiometry (DXA) and the Fracture Risk Assessment Tool (FRAX) [5]. However, while a T-score of BMD below -2.5 increases OVCF likelihood, discordance between BMD and fracture risk is common, as many patients with T-scores above -2.5 still experience fractures [6]. Therefore, it may not be accurate to evaluate the risk of fracture only based on T-score, because BMD can only reflect bone mass, not bone mass and bone microstructure [7]. FRAX, the most widely used assessment tool globally, enhances predictive capability by combining BMD with age and clinical risk factors [8]. Nevertheless, it has some limitations for vertebral fracture prediction [9]. Consequently, there remains substantial potential for refining osteoporotic fracture risk assessment methodologies.

Recent studies on the muscle–bone interaction have provided new perspectives for fracture risk prediction [10, 11]. The paraspinal muscles play a pivotal role in maintaining spinal stability and normal physiologic function [12]. Degenerative changes in these muscles have been confirmed to be closely associated with OVCF occurrence [13-15]. On imaging, paraspinal muscle mass degeneration primarily manifests as reduced cross-sectional area (CSA) and increased fatty infiltration (FI) [16]. Such degenerative changes lead to diminished muscular strength and function, diminishing spinal stability, and increasing vertebral loading. Concurrently, reduced mechanical stimulation from weakened muscle contraction exacerbates bone loss, finally forming a vicious cycle of “muscle degeneration‒bone fragility” [17, 18]. However, current risk assessment tools of OVCF ignore the effect of muscle degeneration.

The purpose of this study is to investigate the clinical value of paraspinal muscle quality alterations (particularly CSA and FI) in OVCF risk assessment, and to evaluate their synergistic utility when combined with conventional indicators (BMD and FRAX scores). Our findings may provide new evidence for the early identification of high-risk populations and provide a reference for the optimization of OVCF risk assessment protocols.

## 2 MATERIALS AND METHODS

### 2.1 Study Population

A total of 258 osteoporosis patients aged ≥ 50 years were retrospectively enrolled from the First Affiliated Hospital of Kunming Medical University (January 1, 2020 – January 1, 2022). After propensity score matching, 129 OVCF patients (fracture group) and 129 non-fracture osteoporosis patients (non-fracture group) were included. The study was approved by the Ethics Committee and followed the ethical standards of the 1964 Declaration of Helsinki.

Fracture group inclusion criteria: (1) Osteoporosis diagnosed by DXA (T-score≤−2.5 at lumbar spine); (2) History of thoracolumbar back pain without or with minor trauma; (3) Imaging evidence of vertebral fracture, such as vertebral height loss, wedge deformity, or bone marrow edema; (4) Exclusion of bone tumors and other pathological fractures; (5) thoracic/lumbar OVCF involving ≥1 vertebral level. Non-fracture group inclusion criteria: (1) osteoporosis confirmed by DXA (T-score≤−2.5); (2) no evidence of vertebral fractures. The exclusion criteria were: (1) Pathological fractures (*e.g*., due to malignancy, infection, or iatrogenic factors). (2) High-energy or burst fractures, or fractures with bone fragments in the spinal canal. (3) Severe lumbar scoliosis (Cobb angle>20°) or spondylolisthesis. (4) History of spinal surgery. (5) Comorbidities or chronic diseases significantly affect bone or endocrine metabolism (*e.g*., diabetes, liver or kidney diseases, cartilage diseases, thyroid disorders, ankylosing spondylitis, diffuse idiopathic hyperostosis, or connective tissue diseases). (6) Treatment with hormonal or anti-osteoporotic drugs. (7) Incomplete clinical data. (8) Images with significant artifacts.

### 2.2 Clinical Data Collection

Relevant demographic and anthropometric information, including age, sex, height, weight, and body mass index (BMI), was extracted from the institutional electronic medical record system. Based on Chinese BMI criteria, participants were categorized into three groups: underweight (BMI˂18.5 kg/m^2^), normal weight (18.5≤BMI˂23.9 kg/m^2^), and overweight (BMI≥24kg/m^2^). When serial admissions yielded discrepant data, we contacted participants by telephone for confirmation.

BMD of the L1-L4 vertebrae was measured using a DPX-L dual-energy X-ray absorptiometer (GE Lunar, Madison, WI, USA). The device automatically generated T-scores according to the standard formula: T-score = (patient BMD - young-adult mean BMD) / young-adult standard deviation.

The 10-year probability of major osteoporotic fractures was calculated with the online FRAX tool (https://frax.shef.ac.uk/FRAX/index.aspx?lang=cht). After selecting the “Asian–China” epidemiological setting, all required variables were entered sequentially, and the platform returned the corresponding FRAX scores.

### 2.3 Imaging Data Collection

All participants underwent lumbar spine magnetic resonance imaging (MRI) using one of the following scanners: Siemens 1.5T, GE Discovery 750W 3.0T, or Philips Achieva 3.0T. Surface coils were utilized during the scans, with patients positioned supine and head-first. For each subject, axial T2-weighted imaging (T2WI) and sagittal T1-weighted imaging (T1WI) and T2WI sequences were acquired (Table **1**).

The severity of OVCFs was assessed using a semi-quantitative grading method [19]. The degree of vertebral compression was determined by comparing the height at the most compressed point of the vertebral body to the posterior height of the same vertebra, or to that of the adjacent superior vertebra in cases of complete collapse. The grading criteria were as follows: grade 1 (mild), 20-25% height loss; grade 2 (moderate), 26-40% height loss; grade 3 (severe), >40% height loss.

The CSA and FI of the bilateral paraspinal muscles (multifidus and erector spinae) were measured by ImageJ software. The regions of interest were outlined at the L3 vertebral body endplate level in the T2 axial image, and the CSA of the region of interest was measured. The CSA was outlined along muscle borders, carefully excluding the supraspinous ligament and osteophytes (Fig. **1a**), and the corresponding grayscale histogram for muscle signal characterization was generated (Fig. **1b**). For fat-threshold standardization, a region of interest was manually drawn in the subcutaneous fat as a reference (Fig. **1c**), and its grayscale histogram was obtained (Fig. **1d**). Using the optimal fat-intensity threshold automatically calculated by ImageJ, threshold-based segmentation was performed to identify the fat-infiltrated area within the paraspinal muscle ROI (Fig. **1e**). The grayscale histograms of the paraspinal muscle ROI and subcutaneous fat ROI were then overlaid to illustrate the threshold-determination process (Fig. **1f**). Two distinct signal-intensity peaks, corresponding to muscle and adipose tissue, were identified, and the overlapping signal-intensity range was defined as the fat-infiltrated area. *FI* was calculated as follows:


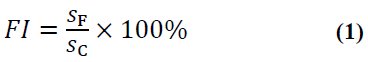


Where *S_F_* denotes fatty infiltrated area and *S*_C_ represents the CSA of the paraspinal muscle, as shown in Fig. (**1a** and **e**).

### 2.4 Statistical Analysis

All statistical analyses were conducted using SPSS (version 27.0; IBM Corp., Armonk, NY, USA). Continuous variables were expressed as mean ± standard deviation or median (interquartile range), depending on whether they followed a normal distribution as determined by the Shapiro–Wilk test. For between-group comparisons, independent samples t-tests were applied for normally distributed data, while the Mann–Whitney U test was used for non-normally distributed data. Categorical variables were reported as numbers and percentages, and group differences were assessed using the χ^2^ test or Fisher’s exact test, as appropriate. The diagnostic utility of T-score, FRAX score, FI, and their combination in evaluating the risk of OVCF was examined by generating receiver operating characteristic (ROC) curves. The area under the curve (AUC), sensitivity, and specificity were computed for all individual parameters. Differences between AUC values were compared using the DeLong test. *P*-value ˂0.05 was considered statistically significant between groups.

Sample size justification: This was a retrospective study; therefore, the sample size was primarily determined by the number of eligible patients during the study period. To justify adequacy for the study objectives, we performed a post-hoc power evaluation based on the primary imaging biomarker (paraspinal FI) and ROC analysis. With 129 OVCF cases and 129 matched controls, the observed between-group difference in FI (29.50 ± 8.56 *vs*. 24.25 ± 5.74) corresponds to a moderate-to-large effect size (Cohen’s d≈0.72), providing>99% power to detect this difference at a two-sided α=0.05. Likewise, a case-control sample of 129/129 provides>99% power to detect an AUC around 0.70 versus 0.50. In addition, the multivariable logistic regression included four key predictors (BMI, FRAX score, CSA, and FI), yielding approximately 32 events per variable (129 events/4 predictors), which exceeds commonly recommended minimums and supports model stability.

## 3 RESULTS

### 3.1 Participant Characteristics

A total of 258 patients were enrolled in this study, comprising 129 fracture cases (31 males, 98 females; mean age 74.05 years) and 129 non-fracture controls (36 males, 93 females; mean age 71.91 years). There were no significant differences between groups in terms of age, sex, or height (*P*>0.05). However, significant differences between groups were found for weight, BMI, FRAX score, T-score, CSA, and FI (all *P*<0.05). Specifically, the fracture group demonstrated significantly lower values for weight, BMI, and CSA, and higher FRAX scores and FI compared to controls (all *P*<0.05). Detailed comparisons of these parameters are presented in Table **2**.

### 3.2 Comparative Analysis across BMI Categories

Because BMI differed significantly between fracture and non-fracture groups, we further examined the relationship between various parameters and OVCF risk within different BMI categories. Participants were stratified by BMI levels (detailed in Methods 2.2). Comparative analysis revealed that FI differed significantly between fracture and non-fracture groups across all BMI categories (*P*<0.05). However, CSA demonstrated significant differences only in the normal weight group (*P*<0.05). No other parameters showed significant differences between groups at any BMI level (Table **3**).

### 3.3 Comparative Analysis by Fracture Severity

To further elucidate the relationship between each parameter and OVCF risk, fracture cases were stratified by severity according to the Genant classification. The cases were divided into non-severe fractures (Genant 1-2, n=74) and severe fractures (Genant 3, n=55). The FRAX score and FI in the severe fracture group were significantly higher than those in the non-severe group (*P*<0.05), while the other parameters showed no significant differences, as shown in Table **4**.

### 3.4 Comparison of FRAX Scores and FI Levels within the Fracture Cohort

Patients in the fracture group were divided into low and high subgroups based on the median of FRAX scores and FI, respectively. Comparative analyses of clinical characteristics and fracture severity across these subgroups are presented in Table **5**.

In the low-FRAX score group, no significant differences were observed between non-severe (Genant 1-2) and severe (Genant 3) fractures regarding age, sex, height, weight, BMI, or CSA (all *P*>0.05). However, severe fractures exhibited significantly higher *FI* levels than non-severe fractures (26.27% *vs*. 22.40%, *P*=0.004), suggesting that FI is a useful indicator of fracture severity in low-FRAX score populations. Conversely, in the high-FRAX score group, none of the evaluated parameters, including FI, differed significantly between non-severe and severe fractures (all *P*>0.05), suggesting that FI has limited ability to discriminate fracture severity in high-FRAX populations. Furthermore, when analyzing low-FI and high-FI subgroups separately, no significant differences were found between non-severe and severe fractures in any of the measured parameters (age, sex, height, weight, BMI, CSA, or FRAX score; all *P*>0.05).

### 3.5 Logistic Regression Analysis of Risk Factors for OVCF

To evaluate the strength of association with OVCF risk, variables significantly different between fracture and non-fracture groups (BMI, FRAX score, CSA, and FI) were entered into univariable logistic regression. All four parameters-BMI (*P*=0.006), FRAX score (*P*=0.004), CSA (*P*=0.007), and FI (*P*˂0.001)-were significantly associated with OVCF Table **6**.

Subsequently, variables with *P* < 0.05 in the univariable analysis were incorporated into a multivariate logistic regression. After adjustment, FI still maintained its significant association (Odds Ratio (OR)=1.091; 95%CI: 1.041-1.144; *P*˂0.001), whereas BMI (*P*=0.137), FRAX score (*P*=0.410), and CSA (*P*=0.096) lost statistical significance. These findings demonstrate that FI is an independent risk factor for OVCF.

### 3.6 Comparative Evaluation of OVCF Risk Assessment Models

We conducted ROC curve analysis to compare the fracture risk assessment capabilities of FI against conventional OVCF risk assessment tools (T-scores and FRAX scores), both individually and in combination. The results showed that FI had a significantly higher AUC (0.702, 95%CI: 0.637-0.767) than T-scores (0.517, 95%CI: 0.445-0.590) and FRAX scores (0.592, 95%CI: 0.521-0.662). Notably, the combined model incorporating all three parameters showed the best fracture risk assessment efficacy (AUC=0.776, 95%CI: 0.719-0.834), with 54.4% sensitivity and 89.7% specificity. DeLong pairwise comparisons confirmed that the combined model significantly outperformed each parameter (all *P*<0.05), whereas no significant difference was observed between T-scores and FRAX scores alone (*P*=0.287). Collectively, FI provides superior risk assessment for OVCF compared to traditional tools. Combining FI with T-score and FRAX score substantially improves risk evaluation accuracy, offering a more reliable method for early identification of high-risk populations in clinical practice. The complete ROC curves and detailed comparisons are presented in Fig. (**2a**, **b** and Table **7**).

## 4 DISCUSSION

This study investigated the clinical value of paraspinal muscle quality changes (mainly including CSA and FI) in OVCF risk assessment and analyzed their combined application effects with conventional indicators (T-score and FRAX score). Through retrospective analysis of clinical and imaging data from 258 patients, we demonstrated that FI possesses significant independent value in OVCF risk assessment, and its combination with traditional indicators substantially enhances OVCF fracture risk evaluation performance.

### 4.1 Association Between Paraspinal Muscle Quality Changes and OVCF Risk

Our findings demonstrate a significant association between lumbar paraspinal muscle FI and OVCF risk, consistent with prior studies [20-22]. The fracture group exhibited markedly higher FI compared to non-fracture controls, with this difference persisting across all BMI categories, most prominently in normal-weight individuals. It is indicated that FI is a BMI-independent metric suitable for universal risk assessment. Previous studies have demonstrated that both low and high BMI predispose to OVCF [23, 24]; therefore, heightened attention to paravertebral FI is especially important in the normal-weight population.

Furthermore, FI correlated not only with fracture presence but also with fracture severity. Patients with severe (Genant 3) fractures displayed significantly greater FI than those with mild-to-moderate fractures, aligning with previous studies [25, 26]. Histologically, FI of paraspinal muscles means that muscle fibers are replaced by adipose tissue. On the one hand, the spine needs to bear greater axial loads, and the vertebral body stress is concentrated. On the other hand, the mechanical stimulation generated by muscle contraction is reduced, which disrupts bone metabolism and exacerbates bone loss, resulting in a vicious cycle of “muscle degeneration‒bone fragility” [27, 28]. Importantly, among individuals with low FRAX scores, FI remained capable of distinguishing severe from non-severe fractures, suggesting that conventional risk models may underestimate fracture risk in patients with pronounced muscle degeneration yet preserved BMD. As an integrated imaging marker of both muscle structure and function, FI effectively fills this assessment gap.

Finally, this study found that FI was an independent risk factor for OVCF. In multivariate logistic regression, only FI (OR=1.091, *P*<0.001) maintained significance, while BMI, FRAX score and CSA did not. This finding also underscores that FI, rather than muscle volume index (CSA), more accurately reflects the true extent of paraspinal muscle mass loss, which is consistent with previous findings [29]. Consequently, FI offers superior independence and clinical utility for fracture-risk prediction.

### 4.2 Comparative Efficacy of FI Versus Conventional Assessment Tools and their Combined Value

The present study further validated the superiority of FI in OVCF risk assessment by ROC curve analysis. FI achieved significantly better performance (AUC=0.702, sensitivity=53.5%) than T-score and FRAX score, which is similar to previous studies. Gnudi *et al*. found that even in patients with osteopenic BMD (T-score>-2.5), elevated fat infiltration in multifidus muscles independently increased vertebral fracture risk [30]. This highlights a critical limitation of BMD, which reflects bone quantity but not bone quality or microarchitecture, and is susceptible to interference from bone tissue degeneration and soft tissue calcifications [31]. Leslie *et al*. similarly noted that FRAX demonstrates limited utility for vertebral fracture prediction [9]. Although the FRAX in combination with BMD effectively forecasts hip fracture risk, its performance for other fragility fractures-particularly OVCF-is complex and limited. It fails to incorporate several crucial predictors of individual fracture risk, such as physical activity, vitamin D deficiency, fall likelihood assessment, and bone turnover markers [32]. Moreover, the original FRAX data are mainly from European and American populations, whereas Asian populations exhibit a markedly higher incidence of OVCF relative to hip fracture [33]. The advantage of FI is that it can reflect changes in muscle mass, which is a key factor in maintaining spinal stability and normal physiological function [11]. As a radiologically visible indicator of muscle-bone crosstalk, FI simultaneously reflects neuromuscular degeneration, microvascular impairment, and metabolic dysregulation, and can capture the high-risk individuals who exhibit microarchitectural bone deterioration while still classified as osteopenic by BMD criteria. Consequently, the diagnostic efficacy of the integrated assessment model constructed by FI in combination with T-score and FRAX score was significantly better than that of a single index. By integrating bone mass (BMD), traditional risk factors (FRAX), and muscle quality (FI), the combined model realized a comprehensive assessment of the overall risk of the “bone-muscle” system, which significantly improved the effectiveness of fracture risk assessment.

FI, as an easily accessible, quantitatively stable index, can compensate for the shortcomings of traditional assessment tools in identifying the risk of “muscle degeneration” fractures, especially in patients with non-significantly reduced T-score or low FRAX score, and has a strong complementary value. In the future, integrating FI into routine OVCF-risk assessment enables more accurate individualized prediction and tailored therapeutic strategies, with the potential to reduce fracture incidence and enhance patient outcomes.

## 5 LIMITATIONS AND FUTURE DIRECTIONS

This study has several limitations that should be acknowledged. Primarily, the retrospective study design is susceptible to both selection and information bias. Second, the limited sample size from a single center restricts the generalizability of our findings. Therefore, validation in larger samples and diverse geographical populations is needed. Additionally, FI is an indicator of muscle quality degeneration, and it can be affected by factors (*e.g*., lifestyle, physical activity level, nutritional status). This study has not yet included these interfering factors for control.

Future studies can be expanded in the following directions: (1) designing prospective, multicenter, large-sample studies to validate the predictive stability of FI in different populations; (2) combining functional assessments (*e.g*., grip strength, balance testing) with inflammatory and metabolic indicators to gain insights into the mechanisms of interactions between FI and bone-muscle health; and (3) interventional trials examining whether targeted exercise or nutritional programmes can reverse FI and, consequently, reduce fracture risk, thereby expanding FI’s role as both a monitoring tool and a therapeutic target.

## CONCLUSION

This study demonstrates that patients with OVCF exhibit markedly deteriorated paraspinal muscle quality, as reflected by reduced CSA and increased FI. Moreover, the level of FI is positively correlated with fracture severity. Furthermore, FI is an independent risk factor for OVCF, and its diagnostic efficacy is superior to that of conventional risk assessment indices. Critically, the integrated model combining FI with T-score and FRAX score significantly improves the efficacy of OVCF risk assessment.

## Figures and Tables

**Fig. (1):**
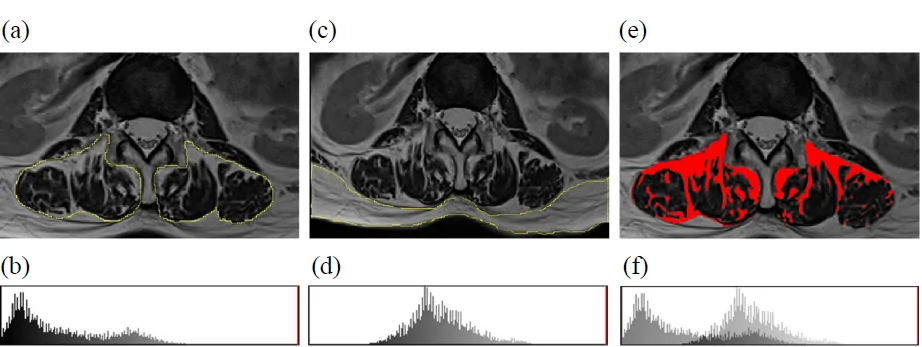
Schematic illustration of paraspinal muscle CSA and FI measurement using ImageJ software. (**a**): Axial T2-weighted image showing manual delineation of bilateral paraspinal muscles; (**b**): Corresponding grayscale histogram for muscle signal characterization. (**c**): Same axial T2WI with a manually drawn region of interest in subcutaneous fat serving as the reference; (**d**): Grayscale histogram of the subcutaneous fat ROI for fat-threshold standardization. (**e**): Thresholded fat-infiltrated areas (red) within the paraspinal muscles, representing quantified FI; (**f**): Overlay of histograms from (b) and (d); ImageJ automatically calculates the optimal fat-intensity threshold.

**Fig. (2):**
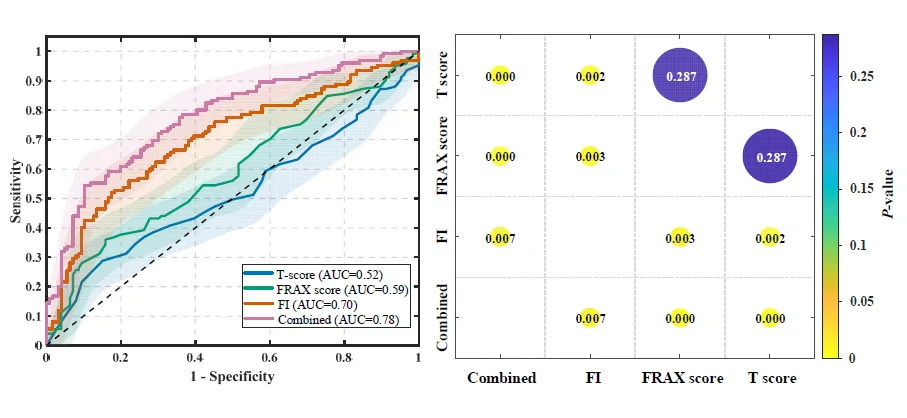
ROC curves and DeLong's test results for T-score, FRAX score, FI, and their combined in assessing OVCF risk. **Note:** In Fig. (**2b**), larger bubble size corresponds to a higher *P*-value (less significant difference) between compared indices. FRAX: Fracture Risk Assessment Tool; FI: fat infiltration.

**Table 1:** MRI scanning parameters.

**Machine**	**Sequence**	**TR(ms)**	**TE(ms)**	**Slice Thickness(mm)**	**Slice Gap(mm)**	**Number of Slices**
Siemens 1.5T	T2WI Sag	3290.0	102.0	4	4	13
	T1WI Sag	350.0	8.5	4	4	13
	T2WI Ax	4160.0	89.0	5	5	20
GE 3.0T	T2WI Sag	2300.0	110.0	4	4	13
	T1WI Sag	480.0	9.4	4	4	13
	T2WI Ax	2960.0	115.3	5	5	20
Philips 3.0T	T2WI Sag	2626.1	120.0	4	4	13
	T1WI Sag	734.7	8.0	4	4	13
	T2WI Ax	3557.3	120.0	5	5	20

**Note:** TR: repetition time; TE: echo time; Sag: sagittal plane; Ax: axial plane; T1WI: T1-weighted imaging; T2WI: T2-weighted imaging.

**Table 2:** Baseline and paraspinal muscle characteristics.

**Characteristic**	**OVCF Group (*n*=129)**	**Non-fracture Group (*n*=129)**	***P*-value**
Age (years)	74.05 ± 9.41	71.91 ± 9.94	0.077
Gender (M/F)	31/98	36/93	0.478
Height (cm)	156.94 ± 8.87	157.89 ± 7.57	0.354
Weight (kg)	54.78 ± 9.56	58.37 ± 8.70	0.002**
BMI (kg/m^2^)	22.25 ± 3.51	23.42 ± 3.15	0.005**
FRAX score	31.30 ±18.43	25.15 ± 13.96	0.003**
T-score	-3.90(-4.60,-2.80)	-3.80(-4.45,-3.10)	0.689
CSA (mm^2^)	3183.11 ±916.45	3476.01 ± 778.64	0.006**
FI (%)	29.50 ± 8.56	24.25 ± 5.74	<0 .001**

**Note:** Data are presented as mean ± standard deviation or median (interquartile range). BMI: body mass index; FRAX: Fracture Risk Assessment Tool; BMD: bone mineral density; CSA: cross-sectional area of paraspinal muscles; FI: fatty infiltration. *P<0.05, **P<0.01 *vs* non-fracture group (statistically significant differences).

**Table 3:** Comparative analysis of fracture and non-fracture groups across BMI categories.

**BMI(Kg/m^2^)**	**Groups**	**Sample** **(n)**	**Age** **(years)**	**Sex** **(M/F)**	**FRAX-score**	**T-score**	**CSA** **(mm^2^)**	**FI** **(%)**
Underweight (BMI<18.5)	Fracture group	16	76.81±8.85	2/14	36.73±19.13	-4.50(-5.20,-2.85)	2750.71±578.16	35.11±9.57
	Non-fracture group	4	81.25±12.18	2/2	24.90±12.39	-4.45(-4.98,-3.83)	3164.78±418.34	22.44±4.39
	*P*-value	-	0.414	0.162	0.262	0.925	0.198	0.020*
Normal weight (18.5≤BMI<23.9)	Fracture group	76	74.28±9.72	24/52	30.34±19.22	-3.90(-4.60,-2.80)	3111.47±897.35	29.15±8.36
	Non-fracture group	77	71.30±10.07	20/57	25.47±13.70	-3.90(-4.60,-3.35)	3534.05±701.54	24.878±5.86
	*P*-value	-	0.065	0.444	0.78	0.402	0.001*	<0 .001*
Overweight (24≤BMI)	Fracture group	37	72.41±8.89	5/32	31.01±16.49	-3.40(-4.30,-2.83)	3517.25±980.16	27.79±7.70
	Non-fracture group	48	72.13±9.36	14/34	24.67±14.74	-3.40(-4.35,-2.80)	3408.85±908.72	23.39±5.61
	*P*-value	-	0.889	0.086	0.069	0.835	0.600	0.005*

**Note:** Data are presented as mean ± standard deviation or median (interquartile range). BMI: body mass index; FRAX: Fracture Risk Assessment Tool; BMD: bone mineral density; CSA: cross-sectional area of paraspinal muscles; FI: fatty infiltration. *P<0.05, **P<0.01 *vs* non-fracture group (statistically significant).

**Table 4:** Comparison of parameters between different fracture severity groups.

**Parameter**	**Non-severe fractures (Genant 1-2)** **(*n*=74)**	**Severe fractures (Genant 3)** **(*n*=55)**	***P*-value**
Age(years)	73.53±9.90	74.76±8.73	0.462
Sex(M/F)	21/53	10/45	0.180
Height(cm)	158.03±10.13	155.47±6.63	0.106
Weight(kg)	56.04±9.45	53.082±9.52	0.082
BMI(kg/m^2^)	22.46±3.42	21.95±3.63	0.416
FRAX score	28.44±15.93	35.14±20.27	0.037*
T-score	-3.70(-4.50,-2.80)	-4.00(-4.70,-2.80)	0.366
CSA(mm^2^)	3228.79±888.74	3121.64±957.26	0.513
FI(%)	27.85±8.89	31.72±7.61	0.010*

**Note:** Data are presented as mean ± standard deviation or median (interquartile range). BMI: body mass index; FRAX: Fracture Risk Assessment Tool; BMD: bone mineral density; CSA: cross-sectional area of paraspinal muscles; FI: fatty infiltration. *P<0.05, **P<0.01 *vs* non-severe fracture group (statistically significant).

**Table 5:** Comparative analysis of fracture severity by FRAX score and FI stratification in osteoporotic fractures.

-	**Groups**	**Sample** **(n)**	**Age** **(years)**	**Sex** **(M/F)**	**Height** **(cm)**	**Weight** **(kg)**	**BMI** **(kg/m^2^)**	**CSA** **(mm^2^)**	**FRAX score/** **FI(%)**
Low-FRAX score	Non-severe fractures	39	72.67±8.91	19/20	160.41±12.44	59.00(52.00, 61.00)	21.64(20.20, 23.73)	3305.38±854.48	22.40(19.53, 28.26)
	Severe fractures	25	73.60±8.10	7/18	156.44±6.57	54.00(49.50, 62.50)	22.59(20.03, 26.61)	3225.71±975.64	26.27(23.11, 33.15)
	*P*-value	-	0.674	0.100	0.147	0.424	0.620	0.732	0.004*
High-FRAX score	Non-severe fractures	35	74.49±10.96	2/33	155.37±5.80	54.46±9.39	22.50±3.30	3143.46±930.38	31.26(29.41, 34.65)
	Severe fractures	30	75.73±9.25	3/27	154.67±6.68	50.80±9.21	21.24±3.60	3034.91±949.44	33.27(28.99, 38.81)
	*P*-value	-	0.625	0.518	0.650	0.119	0.146	0.644	0.236
Low-FI	Non-severe fractures	37	70.90±8.78	14/27	158.00(155.00, 165.00)	59.00(51.50, 61.00)	22.04(20.30, 24.12)	3235.77±957.44	18.00(9.80, 27.00)
	Severe fractures	48	73.83±9.33	6/18	155.50(150.00, 160.00)	54.00(47.75, 63.00)	21.88(20.01, 26.67)	3401.43±890.22	21.00(15.00, 29.00)
	*P*-value	-	0.290	0.441	0.062	0.688	0.935	0.492	0.110
High-FI	Non-severe fractures	-	76.79±10.37	7/26	157.00(152.00, 160.00)	55.27±10.69	22.14±3.49	3088.90(2774.84, 3704.71)	39.26±14.74
	Severe fractures	-	75.48±8.32	4/27	155.00(151.00, 160.00)	51.00±7.91	21.14±3.29	3193.75(2258.01, 3679.49)	43.79±22.09
	*P*-value	-	0.583	0.379	0.328	0.076	0.173	0.432	0.348

**Note:** Data are presented as mean ± standard deviation or median (interquartile range). BMI: body mass index; FRAX: Fracture Risk Assessment Tool; BMD: bone mineral density; CSA: cross-sectional area of paraspinal muscles; FI: fatty infiltration. *P<0.05, **P<0.01 *vs* non-severe fracture group (statistically significant).

**Table 6:** Univariate and multivariate logistic regression analysis for OVCF risk.

**Variables**	**Univariate Logistic Regression Analysis**		**Multivariate Logistic Regression Analysis**	
	**OR (95% CI)**	***P*- value**	**OR (95% CI)**	***P*-value**
Age	1.023(0.997,1.050)	0.080	-	-
Sex	0.817(0.468,1.417)	0.478	-	-
BMI	0.899(0.832,0.970)	0.006*	0.938(0.863,1.020)	0.137
FRAX score	1.024(1.007,1.040)	0.004*	1.008(0.989,1.028)	0.410
CSA	0.695(0.534,0.960)	0.007*	0.784(0.588,1.044)	0.096
FI	1.114(1.069,1.161)	˂0.001**	1. 091(1.041,1.144)	˂0.001**

**Note:** BMI: body mass index; FRAX: Fracture Risk Assessment Tool; CSA: cross-sectional area of paraspinal muscles; FI: fat infiltration; OVCF: osteoporotic vertebral compression fracture. *P<0.05, **P<0.01.

**Table 7:** ROC curve analysis of T-score, FRAX score, FI, and their combined use for assessing OVCF risk.

**Parameter**	**AUC**	**95%CI**	**Sensitivity (%)**	**Specificity (%)**
T-score†	0.517	0.445~0.590	27.9	85.3
FRAX score†	0.592	0.521~0.662	36.0	84.1
FI†	0.702	0.637~0.767	53.5	82.2
combined	0.776	0.719~0.834	54.4	89.7

**Note:** AUC: area under the curve; 95% CI: 95% confidence interval; FRAX: Fracture Risk Assessment Tool; FI: fat infiltration. †: *P* < 0.05 *vs*. combined model (DeLong's test).

## Data Availability

The datasets used and/or analyzed during the current study are available from the corresponding authors [L.Y] and [Y.H] upon reasonable request.

## LIST OF ABBREVIATIONS

AUC: Area under the curve
FRAX: Fracture risk assessment tool
BMD: Bone mineral density
MRI: Magnetic resonance imaging
BMI: Body mass index
OVCF: Osteoporotic vertebral compression fractures
CSA: Cross-sectional area
OR: Odds ratio
CI: Confidence interval
T1WI: T1-weighted imaging
DXA: Dual-energy X-ray absorptiometry
T2WI: T2-weighted imaging
FI: Fat infiltration
ROI: Region of interest

## STANDARDS OF REPORTING

STROBE guidelines were followed.
